# General Practitioners’ Knowledge, Attitudes, and Practices of Dietary Advice for Weight Control in Their Overweight Patients: A Scoping Review

**DOI:** 10.3390/nu15132920

**Published:** 2023-06-27

**Authors:** Hilmi S. Rathomi, Tanya Dale, Nahal Mavaddat, Sandra C. Thompson

**Affiliations:** 1 School of Population and Global Health, University of Western Australia, Crawley, WA 6009, Australia; 2 Faculty of Medicine, Universitas Islam Bandung, Bandung 40116, Indonesia; 3 Western Australian Centre for Rural Health, University of Western Australia, Geraldton, WA 6530, Australia; 4 UWA Medical School, University of Western Australia, Crawley, WA 6009, Australia; 5 School of Allied Health, University of Western Australia, Crawley, WA 6009, Australia

**Keywords:** dietary advice, GPs’ knowledge, attitude, practice, weight control

## Abstract

This scoping review assessed the knowledge, attitudes, and practices of general practitioners (GPs) regarding dietary advice for weight management. A systematic search of PubMed, EMBASE, CINAHL, and MEDLINE was conducted for any qualitative, quantitative, and mixed-methods studies published in the past five years that informed GPs’ dietary advice for weight control. Thirteen studies were included in the analysis after screening 881 papers. These studies tended to focus mostly on GPs’ practices rather than their knowledge and attitudes. The most frequently mentioned dietary advice was to reduce calorie intake; however, 32 different types of dietary advice were identified in the literature, including approaches such as intermittent fasting and a ketogenic diet that are not recommended in current guidelines. GPs showed varying levels of knowledge and attitudes regarding the best dietary advice for patients. Further research is needed to better understand GP perspectives, with efforts to assist GPs in providing tailored advice based on the latest evidence to improve patient outcomes required.

## 1. Introduction

Weight-related issues have become a growing global concern, with approximately two billion individuals worldwide being either overweight or obese. This condition significantly increases their risk for major diseases, including heart disease, stroke, diabetes, and cancer [1]. Urgent solutions are required to mitigate this trend. While pharmacological and surgical approaches to obesity have advanced significantly, lifestyle modifications remain the primary approach to treatment [2]. The current widely adopted primary strategy for weight control emphasizes the importance of achieving a calorie deficit underpinned by eating less and moving more [1,2].

Emerging evidence, however, suggests that when it comes to diet, relying solely on a calorie deficit may not be sufficient for long-term weight control [3]. Maintaining a calorie deficit is often challenging and can result in a reduction in metabolic rate, which may lead to weight regain [4,5]. Moreover, it is now recognized that the types and timing of food intake may have different metabolic effects on the body and influence the effectiveness of weight loss efforts [6,7,8]. Additionally, alternative dietary approaches with less certain evidence, such as the ketogenic diet, intermittent fasting, the paleo diet, the Mediterranean diet, and others, have gained popularity and recommendations based on the evidence and are being considered for inclusion into guidelines by some professional associations [9,10,11].

General practitioners (GPs) play a crucial role in guiding patients’ health behaviors, including advising on weight loss. GPs have access to a variety of lifestyle, medical, and surgical approaches to weight loss to assist their patients. However, patients may also have access to numerous diets that have gained ground from various sources, some of which may be promoted by GPs [12], in addition to the advice provided by established guidelines. Many people endeavor to lose weight by following advice from the Internet, friends, and family [13], often without long-term success. GPs frequently express uncertainty and frustration regarding the most effective methods for assisting patients with obesity in their weight loss efforts based on available guidelines [14].

This literature review explored the dietary advice provided by GPs to their overweight patients requiring weight loss to help identify and fill the gap in understanding the real-world practices of GPs. A 2019 systematic review identified the need for more research to understand the specific weight loss advice given by primary care practitioners to their patients [15]. By exploring GPs’ knowledge, attitudes, and practices regarding dietary advice for weight loss, this review examines the alignment between dietary guidelines and the advice given by GPs. Additionally, it sheds light on the influence of emerging dietary approaches and identifies areas where further research is needed.

In this review, we categorized dietary advice given by GPs based on the definition of dietary patterns. This definition comprises the quantities, proportions, variety, and combination of different foods, drinks, and nutrients in prescribed diets, and the frequency with which they are consumed [16]. It is common to categorize dietary advice into the quantity, quality, and timing of food intake [17]. We classified the advice aimed at reducing total energy intake as “quantity”, while that related to changing the type of foods consumed was classified as “quality”. Advice pertaining to the modification of the proportion of food groups in each meal, such as the Mediterranean diet, was also classified as “quality”, as this dietary approach does not explicitly aim to reduce the number of calories consumed. Recent evidence suggests that the timing of food intake can be important, including the eating duration and timing of meals throughout the day [17]. Hence, any advice related to the frequency, exact timing, or duration of food intake was included in the category of “timing of food intake”. Furthermore, we classified advice related to adherence to dietary guidelines as a separate category, since it involves both the quantity and quality of food consumed. By classifying dietary advice in this manner, we gained a better understanding of how GPs provide dietary recommendations to manage their patients’ weight.

## 2. Materials and Methods

### 2.1. Eligibility Criteria

Our review protocol was based on the JBI guidelines for scoping reviews [18]. We did not register the protocol to PROSPERO since it did not meet the registration requirements. The underpinning question of the review relates to what dietary advice GPs give their patients for managing weight and, more specifically, what GPs’ knowledge, attitudes, and practices are regarding dietary advice for weight management.

We used the SPIDER framework (sample, population of interest, design, evaluation, and research type) to determine eligible studies [19]. This approach is appropriate for the exploratory nature of this review. Search terms used for the spider search are shown in Table 1.

### 2.2. Search Strategy and Article Selection

We conducted a systematic search of the literature using a comprehensive search strategy developed in consultation with two academic librarians (see Supplementary Table S1). The search strategy involved three main concepts: “nutrition advice”, “general practitioners”, and “knowledge attitudes and practices”. We combined the search terms from each concept using the Boolean operator “AND”. We also adjusted the search terms for different search methods with each database.

We searched articles using four databases: PubMed, CINAHL, Ovid EMBASE, and Ovid MEDLINE. All articles published in the last 5 years (1 January 2017—search date) were included. All database searches were carried out on 31 July 2022.

Inclusion and exclusion criteria are shown in Box 1. We included only studies reporting in English, primary research, studies with GP participants, reports about the content/nature of dietary advice, and those which measured the knowledge, attitudes, or practices of GPs.

Box 1 **Inclusion and Exclusion Criteria.**

Inclusion criteria

- Location/Population: international/worldwide
- Target age group: Adults (aged 18 years and above)
- Primary research studies
- Papers published from 1 January 2017 to search date (31 of July 2022)
- Published in English
- Full text available
- All types of study: surveys, trials, cohorts, interviews, focus groups.

Exclusion criteria were as follows:

- Advice for children and adolescents (<18 years old) or pregnancy
- Nutrition problems other than being overweight/obese
- Articles focused on health advice other than nutrition (e.g., exercise, sleep, etc.)
- Editorials, commentaries, theses, book chapters, reviews, systematic reviews
- Grey literature

All articles from each database search were imported into EndNote, and duplicate articles removed, with remaining articles uploaded into Covidence. Eligibility criteria were applied to assess the articles. Two reviewers (H.S.R. and T.D.) screened all references independently in two stages, initially reviewing the title and abstract, and in the second stage reviewing the full text. Any conflicts were resolved by a third reviewer (S.C.T.) through discussion to reach a consensus.

### 2.3. Data Charting Process and Synthesis

All members of the research team agreed upon the Microsoft Excel (Microsoft Corporation, Redmond, WA, USA) extraction spreadsheet. Information from included studies was captured independently by H.S.R. and T.D. with a data extraction tool that was modified iteratively based on discussions (Supplementary Table S2).

Results are described in a narrative synthesis which did not discriminate between studies based on their quality and was guided by the PRISMA-ScR checklist (Supplementary Table S3) [20].

## 3. Results

### 3.1. Selection of Sources of Evidence

**Figure 1:**
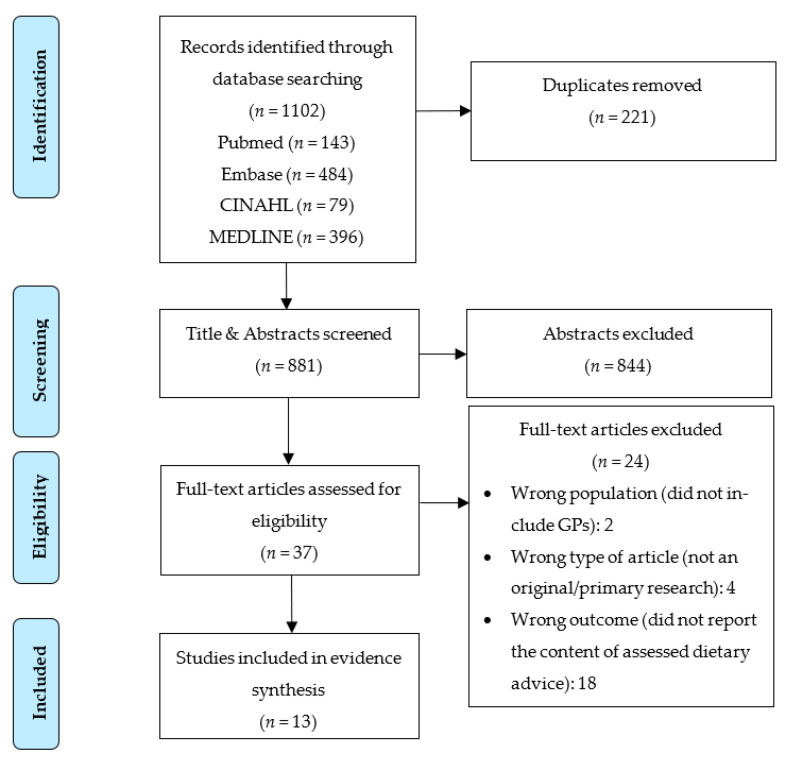
PRISMA diagram of study selection process.

The database search and screening process (Figure 1) initially identified 1102 journal articles. After the removal of 221 duplicate publications, 881 studies underwent abstract and title screening. We excluded 844 studies that were not relevant, and 37 studies were assessed for eligibility by full-text review. Of these, 24 studies were excluded due to “wrong outcomes” because the content of dietary advice was not reported (18), being the wrong type of article (4), and because the population being considered was not GPs (2). Thirteen studies were included in the final analysis.

### 3.2. Characteristics of Sources of Evidence

Out of the 13 studies included, the majority were conducted in North American countries (U.S.A. and Canada); no studies were reported from Africa. While only two studies focused solely on GPs [21,22], the remaining eleven studies examined a wide range of specialties or professions, including internal medicine specialists, those working in pediatrics, and nutritionists. However, these studies did not provide separate analyses by the discipline of their health professional participants. Ten studies (77%) were quantitative and relied on self-report surveys through online questionnaires developed by each research team. The sample sizes of the quantitative studies ranged from 38 to 1510 respondents, with response rates ranging from 9 to 81%. On the other hand, the three qualitative studies used in-depth interviews with 6, 20, and 26 participants each. Most studies focused on the practice of GPs, rather than their knowledge or attitudes. Table 2 summarizes the key characteristics of each study and Table 3 provides a summary of each article included.

### 3.3. Content of Dietary Advice Reported

We found that the majority of studies investigated general dietary advice for weight loss, while two studies focused on specific areas of diet, such as sugar-sweetened beverages [32] and added sugar [27]. We identified 32 different types of dietary advice across all studies, with the most common being reduction in calorie intake and elimination diets (each mentioned in 4 articles). When further classifying the advice by dietary aspects such as quantity, quality, timing of food intake, and adherence to guidelines, we found that advice regarding food quality was the most frequently reported topic, with a wide range of approaches. This category encompassed 28 types of advice, including Mediterranean diets [12,23], whole foods [12,24], increasing fruits and vegetables [12,24,30], limiting added sugar [27,32], macronutrient modification [29], and reducing fat consumption [12,29,30].

**Figure 2:**
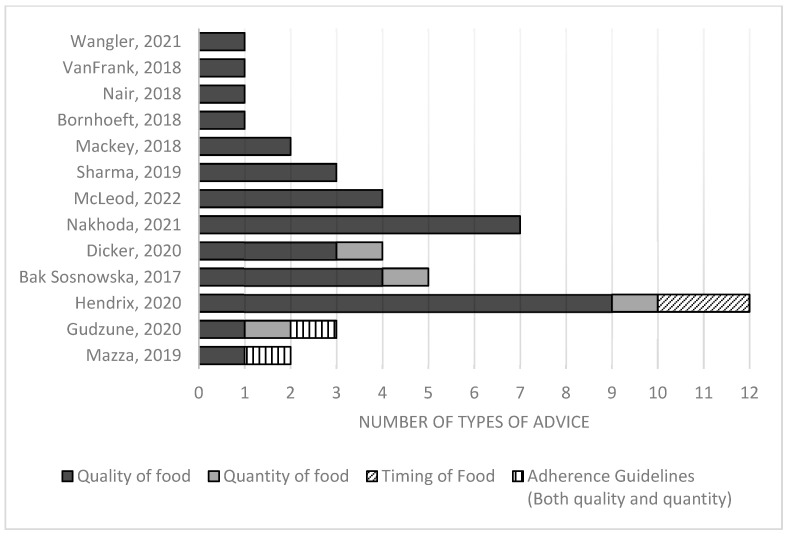
Number of different types of dietary advice reported in each article, classified by whether the advice was related to food quality, quantity, timing, or adherence to guidelines [12,21,22,23,24,25,26,27,28,29,30,31,32].

Only one article reported advice regarding the timing of food intake [12]. This article also discussed many of the latest popular dietary approaches, including ketogenic diets, intermittent fasting, paleo, and vegan diets. Only two articles discussed adherence to guidelines [26,28]. Figure 2 illustrates the dietary advice reported in each eligible study.

#### 3.3.1. Knowledge of GPs

Although knowledge around dietary advice by GPs could be considered fundamental to attitudes and practices, only 5 out of 13 papers discussed GPs’ knowledge regarding dietary guidelines and best practice. In those that examined the knowledge of GPs, many found poor self-ratings in the area of dietary advice. For example, in a survey of 356 physicians from academic and community hospitals in the U.S., of whom 22.3% were family medicine specialists, McLeod et al. found that physicians had varying levels of knowledge regarding different dietary approaches and generally rated their own knowledge in this area as fair to poor. While 59% had good knowledge of the “portion control” approach to dietary weight loss, lower proportions of physicians reported good knowledge regarding other kinds of dietary advice, including the DASH diet (40.3%), macronutrient modification (24.7%), and saturated fat reduction (13.7%) [29]. In another study, Nakhoda et al. also reported that 80% of GPs in Iran felt they had limited nutritional knowledge [30]. Using an exploratory approach, Mazza et al. also reported that many GPs in Australia lacked familiarity with the NHMRC (National Health and Medical Research Council) obesity guidelines, including the dietary aspects advised in those guidelines, with one GP interviewed reporting their confusion about which diets to advise due to the abundance of conflicting information available [28].

#### 3.3.2. Attitudes of GPs

Six papers were reviewed on the attitudes of GPs, including attitudes towards several different dietary approaches [24,25,27,29,30,31]. An international survey project, ACTION-IO (Awareness, Care, and Treatment in Obesity management—An International Observation), found that 84% of healthcare practitioners in Israel agreed that reducing calories is effective, while 38% and 46% agreed on elimination diets and specific diets, respectively, as being effective [25]. However, in Canada, healthcare practitioners believed that general improvement in eating habits was the most effective management approach, with lower proportions of positive attitudes towards elimination diets and specific diet programs (29% and 17%, respectively) [31].

An Iranian-based study of 500 physicians found that GPs had mixed attitudes and beliefs on various strategies to managing metabolic syndrome, including regarding weight reduction. About 21% agreed on the effectiveness of increasing vegetable and fruit intake, 72% on limiting starchy vegetables, and 82% on consuming a variety of whole grains. All (100%) agreed on limiting high-cholesterol foods and 97% on limiting high-fat dairy products [30]. When discussing the topic of added sugar, the physicians generally believed excessive consumption of added sugar significantly contributed to weight gain [27].

#### 3.3.3. Practices of GPs

The practices of GPs in giving dietary advice were addressed in the majority of the articles reviewed (85%). Hendrix et al. conducted a survey of 1151 physicians in the U.S. (17.5% of whom were family practice members) who were part of a Facebook group. The survey asked about their dietary weight-loss strategies and what they recommended to their patients. The study discovered that a wide variety of dietary advice was recommended, with 21–35% of physicians advising intermittent fasting, 25–41% recommending a ketogenic diet, 30–40% suggesting low-carbohydrate calorie restriction, and 17–22% advising a Mediterranean diet [12]. In contrast, from an exploratory approach, Wangler et al. found that GPs recommended a healthy high-fiber diet and combined it with plenty of exercise to achieve successful outcomes for their patients [22].

Gudzune et al. examined 494 physicians who were diplomates of the American Board of Obesity Medicine (ABOM) to evaluate their adherence to multiple guidelines, including the American Heart Association (AHA), the American Association of Clinical Endocrinologists (AACE), the Obesity Medicine Association (OMA), and the Endocrine Society. They found that OMA guidelines had the highest adherence rate (65.6%), followed by AACE with 33%, the Endocrine Society with 31.6%, and the AHA with 30.8% [26]. Another study exploring adherence to guidelines was a qualitative study in Australia, which reported that GPs tried to follow what they remembered from the NHMRC guidelines and combined this with their own personal experience, such as advising their patients to cut down on certain foods [28].

VanFrank et al. conducted a more specific study focusing on GPs’ counseling related to sugar-sweetened beverages (SSBs) for weight control. Of the 1510 U.S. physicians involved (35.9% of whom were family practice members), almost all (98.5%) reported counseling patients on SSBs, including the calorie content of SSBs, added sugar, and advice about reducing the frequency of SSB consumption [32]. This finding is similar to that of Mackey et al., who also reported that 97% of family physicians advised against sugary beverage consumption and 82% advised limiting added sugar in food [27].

## 4. Discussion

Our study aimed to investigate the dietary advice provided by GPs to their patients for weight control, as well as their knowledge, attitudes, and practices in this area. A previous systematic review has shown that this topic is rarely discussed in depth [15]. Moreover, prior studies have mainly focused on the barriers and facilitators of nutrition counseling without providing detailed information on the specific dietary advice given [33]. These gaps emphasize the need to gain a better understanding of the actual dietary practices of GPs.

We identified 32 distinct types of dietary approaches, with some already included in established guidelines, while others were not. The dietary approaches were categorized into four main categories: quality, quantity, timing, and adherence to guidelines. This classification provides valuable insights into the dietary advice offered by general practitioners (GPs). It is noteworthy that while all the studies covered a wide range of advice pertaining to quality, less than half of them specifically addressed the quantity or the importance of reducing calorie intake, which is considered a crucial aspect of weight loss approaches. This suggests a potential shift in focus towards questions around what to eat rather than solely how much to eat. Recognizing the variety of dietary advice given by GPs to patients for weight control highlights the importance of the need for a more standardized approach in this area. Furthermore, only a limited number of studies reported advice regarding adherence to guidelines and the timing of food intake.

Importantly, our findings suggest that GPs may not always follow the guidelines. One study conducted by Hendrix et al. discovered various recent dietary approaches, including intermittent fasting, the ketogenic diet, the Mediterranean diet, and vegan diets to be frequently advised by GPs, with GPs more likely to advise dietary measures which they had personally found effective. This study reported 14 different approaches, accounting for 50% of all identified weight control approaches [12]. Hendrix’s work provides valuable insights into emerging dietary trends considered by GPs and suggests that some GPs deviate from current dietary guidelines for weight loss. This lack of adherence to current dietary guidelines likely reflects GPs’ uncertainty regarding the effectiveness of these dietary approaches [34]. This may be in response to past failures in helping patients with losing weight when applying current guidelines and could explain why GPs are reluctant to provide weight counseling or do not prioritize this for their patients [34].

Our review also revealed several other obstacles for GPs in providing adequate nutritional advice. Inadequate nutrition training during undergraduate and graduate medical education and a resulting lack of confidence and skills in assisting patients with weight loss were reported by GPs as a significant challenge to providing dietary advice [21,30]. Furthermore, time constraints and patient disinterest in weight loss were identified as additional barriers [28,31]. Enhancing GPs’ perceptions of obesity as a significant medical issue, irrespective of the presence of other chronic diseases, could help prioritize important conversations around weight with overweight patients [34]. The presence of a dietitian as part of the primary care team can alleviate time pressure and ensure that patients receive comprehensive weight-loss support and is frequently much valued by GPs in their practice [35].

### Strengths and Limitations

This review is the first to provide a comprehensive range of reported dietary advice to date. The only previous study to explore health advice for weight loss was conducted by Walsh et al. in 2019 [15], who concluded that very few studies document the details of the advice given to overweight patients by GPs to help them lose weight.

It is important to interpret the results of this review of the knowledge, attitudes, and practices of GPs with caution because only studies that explicitly reported the content of advice were included. Several studies were excluded for reasons such as discussing only perceived barriers to giving advice, experiences and timing of dietary counseling, and knowledge and attitudes without providing details on the actual advice given. Additionally, many studies included a variety of health professionals, and separate results were not presented for GPs, emphasizing the need for careful interpretation. Furthermore, the studies included in this review were limited in their generalizability, as they were conducted in specific locations and may not be representative of GPs in other regions.

Indeed, drawing definitive conclusions about the level of knowledge and the attitudes of GPs is challenging due to the heterogeneity of the papers and the use of different instruments. In the course of our review, we discovered that there were no standardized questionnaires used in the studies, except for the ACTION-IO studies reported in Dicker et al. [25] and Sharma et al. [31]. This lack of standardization poses a challenge in comparing the knowledge and practices of GPs across different regions. We suggest developing a standardized questionnaire that includes both advice within established guidelines and open-ended questions to explore dietary advice given outside of these guidelines. This will facilitate meaningful comparisons in the current practices of GPs across different regions, which was not possible in this review. Furthermore, future research should focus on assessing the knowledge, attitudes, and practices of GPs regarding specific diets, particularly within specific populations and in regions lacking evidence, such as Africa.

Finally, we followed the requirements for a scoping review [18] and did not undertake a critical appraisal or quality assessment of the individual articles included. However, in the absence of a formal quality assessment, it is worth noting that the included studies in this scoping review exhibited a diverse range of study designs, methodologies, and limitations. The quantitative studies predominantly relied on self-report surveys through online questionnaires. This method may be limited in capturing the full scope of GPs’ perspectives.

## 5. Conclusions

Our review revealed mixed knowledge and attitudes among GPs regarding various weight management strategies. General practitioners provided a diverse range of dietary advice to their patients, including some advice that is not currently recommended in the guidelines, such as the ketogenic diet, low-carbohydrate calorie restriction, and intermittent fasting. These findings underscore the need to address the deviation of some GPs from the guidelines, with further research needed to understand the reasons behind this and to explore any potential lack of adherence to established guidelines on patient outcomes. One potential reason why GPs may not always follow guidelines is the multitude of differing guidelines and diets that are currently promoted. GPs require consistent, up-to-date guidelines that are effective in helping their patients lose weight. A lack of general nutritional knowledge may also play a role. Interventions are needed to enhance nutritional education for GPs, ensuring that they possess the necessary knowledge and skills to provide dietary advice.

To improve patient outcomes, it is also important to develop living guidelines and make GPs aware of them [36]. Living guidelines aim to provide “ready-to-go” evidence summaries, ensuring access to rigorous and up-to-date evidence while waiting for major guideline revisions [36]. This approach addresses the rapidly evolving evidence in the field, enabling GPs to provide the best possible advice to their patients.

It is, however, crucial to recognize that a one-size-fits-all approach to weight loss may not be effective for all patients. GPs should have access to updated guidelines but should have the scope to provide tailored advice that considers the patient’s unique needs and preferences to enhance patient outcomes.

## Figures and Tables

**Table 1:** Search strategy in SPIDER framework.

Component	Concept
S (sample)	General practitioners (GPs)
P of I (phenomenon of interest)	Dietary advice for weight loss
D (design)	Survey/Interview/Focus groups
E (evaluation)	Knowledge, attitude, and practice
R (research type)	Qualitative, quantitative, and mixed-methods

**Table 2:** Summary of eligible studies’ characteristics (*n* = 13).

Study Characteristics	*n*	Study Characteristics	*n*
*Year of Publication*		*Methods*	
2017	1	Quantitative	10
2018	4	Qualitative	3
2019	2		
2020	2	*Disciplines Included*	
2021	3	Mixed	11
2022	1	General/family practice only	2
*Region*		*Area of Assessment Discussed*	
North America	8	Knowledge	5
South America	0	Attitudes	6
Europe	2	Practices	11
Asia	2		
Africa	0		
Australia	1		

**Table 3:** Summary of studies.

No.	First Author, Year	Aim/Objective	Location/Setting/Design	Participants	Dietary Advice Reported	Dietary Aspect Discussed	Findings
1	Bak-Sosnowska, 2017 [23]	To evaluate GPs’ attitudes towards health and to determine factors affecting diagnosis of obesity in their patients.	Poland Primary care in a refresher course Survey	250 primary care (34% family doctors, 66% other specializations). Age: 25–65 years, mean age 53.6 years, average time since graduation 28 years, 84% women, 87% lived in city.	Mediterranean diet Low-glycemic index Reduce calorie intake Cambridge plan (very low calorie)	Quantity, Quality	Practice: 29.2% always giving advice about diet, 42.5% advised Mediterranean diet, 15.2% on glycaemic index, 4.4% Cambridge diet, 27.2% reducing calorie intake, 4% infusible diet, 6% hunger diet.
2	Bornhoeft, 2018 [24]	To explore perceptions, attitudes, and behaviors toward obesity management by providers in primary care.	U.S. Primary care practices Qualitative	12 PCPs, 6 physicians, and 6 nurses. Inclusion: >1 year experience and see obese patients regularly. 70% women, 83% Caucasian, 67% had >10 years’ experience, aged 30–50 years.	Eat fruits and vegetables Shop at wholefoods	Quality	Practice: GPs said they tell people to eat fruit and vegetables and shop at Whole Foods and Trader Joes.
3	Dicker, 2020 [25]	To identify perceptions, attitudes, behaviors, and barriers to effective obesity treatment among people with obesity (PwO) and physicians in Israel.	Israel Health care practitioners (part of ACTION-IO) Survey	169 healthcare practitioners (HCPs), including physicians, specialists, dieticians, pharmacists, nurses, diabetes educators. Inclusion: in practice for >2 years, at least 50% spent in direct care, had seen >100 patients in the past month, at least 10 patients with high BMI.	Reducing calories Specific diet (unexplained) Elimination diet	Quantity, Quality	Attitude: 84% agreed that reducing calories is effective, 38% agreed on specific diet, 46% agreed on elimination diets. Practice: 62% advised reducing calories, 35% specific diet, 47% elimination diet.
4	Gudzune, 2021 [26]	To determine the clinical services offered by the American Board of Obesity Medicine (ABOM) diplomates and whether guideline-concordant services varied by clinical practice attributes.	U.S. ABOM diplomates Survey	494 ABOM diplomates physicians (response rate 19.2%). 37.5% practiced in urban areas, 11.3% in rural areas.	Adherence to guidelines: AHA/AACE/OMA/Endocrine Society	Guidelines	Practice: 30.8% of services offered aligned with AHA guideline, 33.4% with AACE, 65.6% with OMA, 31.6% with Endocrine Society, and 82.6% with any guideline used.
5	Hendrix, 2020 [12]	To examine the personal dietary weight-loss strategies of female physicians and what they recommended to their patients.	U.S. Physicians in Facebook-based group Survey	1151 participants, members of “Women Physicians Weigh in” Facebook group. (Response rate 9%). Mean age: 40.2, 17.5% family medicine specialists, 81.9% white ethnicity, 56.5% 6–12 months in group.	Intermittent fasting Ketogenic diet Low-carbohydrate calorie restriction Prolonged fasting Mediterranean diet Paleo diet Vegan/vegetarian diet Whole30 diet Low-fat calorie restriction Very low caloriesDASH	Quantity, Quality, Timing	Practice: 21–35% advised intermittent fasting, 25–41% advised ketogenic diet, 30–47% low-carbohydrate calorie restriction, 1–2% prolonged fasting, 16–27% referred to commercial program, 17–22% advised Mediterranean diet, 5–11% paleo, 1–4% vegan, 6–13% Whole30, 3–4% low-fat calorie restriction, 1% very low calories, 8–58% DASH, 3–23% diabetes prevention program.
6	Mackey, 2018 [27]	To describe the knowledge, attitudes, and behaviors of family physicians regarding added dietary sugar.	U.S. Members of major family medicine organizations Survey	1196 family physician members of the council of academic family medicine organizations.	Limit sugary beverages Avoid addition of sugar to foods	Quality	Knowledge: 15% were very familiar with research relating to added sugar, 30% were familiar with guidelines pertaining to added sugar consumption. Attitude: Majority believed excess added sugar contributed to excess body weight. Practice: 72% reported providing dietary counseling to the majority of patients. 97% advised against sugary beverage consumption and 82% advised limiting added sugar in food.
7	Mazza, 2019 [28]	To identify the views of GPs and general practice staff regarding barriers and enablers to implementation of obesity guideline recommendations in general practice.	Australia GP clinics Qualitative	20 GPs and 18 practice staff (14 female).	Implementation of NHMRC guideline Cut down certain foods	Quality, Guidelines	Knowledge: Most had no knowledge on guidelines, six unsure if they had read the guidelines. Practice: One GP advised patients to reduce certain foods.
8	McLeod, 2022 [29]	To investigate primary care physicians’ current knowledge and opinions regarding the delivery of dietary interventions. This work aimed to identify modifiable barriers to prescribing dietary interventions to prevent and treat diet-related diseases.	U.S. Academic and community hospitals Survey	356 physicians (response rate 23%). 62.3% female, 75.5% non-Hispanic white, 56.7% fewer than 10 years’ experience, 22.3% family medicine, 56.7% aged 40 or younger.	DASH diet Portion control Macronutrients content Keto/Saturated fat	Quantity, Quality	Knowledge: 40.3% had good/excellent knowledge of DASH, 59% on portion control, 24.7% on macronutrients, and 13.7% on saturated fat.
9	Nair, 2018 [21]	To examine physician weight-loss nutrition counseling among family physicians in Huntington, West Virginia, an area with the highest obesity prevalence in the United States.	U.S. Ambulatory practice Survey	38 physicians (response rate 81%). 55% were between 35 and 55 years old, 53% men, listed in family medicine section in West Virginia, at least one practice site.	USDA My plate	Quantity, Quality	Practice: 18% using USDA MyPlate resources.
10	Nakhoda, 2021 [30]	To investigate the nutritional knowledge, attitudes, and practices of general physicians (GPs) toward the management of MetS.	Iran Health centers affiliated to university Survey	500 physicians. Mean age: 42.8 years, 58% female.	Consume a variety of fruits and vegetablesVariety of whole-grains Fat-free and low-fat dairy Replace high-fat meat, red meat, and processed meat with fish, legumes, poultry, and lean meats Limit salt to less than 6 g/day Limit high-cholesterol foods (based on guidelines of METs)	Quality	Knowledge: 80% felt they had limited nutritional knowledge. Attitude: Agreement on: low sodium diet (21%), vegetables and fruits (21%), limiting starchy vegetables (72%), consuming high grain foods including refined grain (82%), limiting high-cholesterol food (100%) and high-fat dairy products (97%), replacing red meat with legumes (95%). Practice: Over half recommended reducing cholesterol intake, consuming low-fat dairy. 30% recommended consumption of a variety of grain products, fruits, vegetables; <25% recommended replacing high fats with fish/legume and reducing salt intake.
11	Sharma, 2019 [31]	To investigate perceptions, attitudes, and perceived barriers to obesity management among Canadian people with obesity (PwO), healthcare providers (HCPs), and employers.	Canada Physicians (part of ACTION-IO) Survey	395 HCPs (including physicians, specialists, dieticians, pharmacists, nurses, diabetes educators) (response rate 34%).	General improvement in eating habits Elimination diets Specific diet program (unexplained)	Quantity, Quality	Attitude: 63% believed their role was to encourage general improvement in eating habits, 29% believed in elimination diets, 17% believed in a specific diet or diet program.
12	VanFrank, 2018 [32]	To explore SSB-related topics physicians discuss when counseling overweight/obese patients and examine associations between physicians’ SSB-related counseling and their personal and medical practice characteristics.	U.S. Physicians who participated in WorldOne’s medical panel Survey	1510 physicians currently practicing in the U.S. Inclusion: actively seeing patients, at least 3 years of practice. 52.5% <45 years, 68.2% male, 60.9% non-Hispanic white, 35.9% family practice.	Frequency of sugar-sweetened beverage consumption Calorie content of SSBsAdded sugar in SSBs	Quality	Practice: 98.5% reported counseling patients on SSB, 63% advised about calorie content in SSBs, 53.1% advised about added sugars in SSBs, 63.8% advised about frequency of SSBs.
13	Wangler, 2021 [22]	To explore GPs’ attitudes and behaviors towards obese patients, willingness to provide care, approaches and strategies, and challenges experienced.	Germany GP clinic Qualitative	36 GPs.	Healthy high-fiber diet	Quality	Practice: One GP mentioned that exercise combined with healthy high-fiber diet was reported to bring successful outcomes. (Results about attitudes in this paper were not related to specific approach, so were not included in the extraction)

Abbreviations: PwO: People with obesity, SSB: sugar-sweetened beverages, MetS: metabolic syndrome, AHA: American Heart Association, AACE: American Association of Clinical Endocrinologists, OMA: Obesity Medicine Association, NHMRC: National Health and Medical Research Council.

## Data Availability

No new data were created.

## Supplementary Materials

The following supporting information can be downloaded at https://www.mdpi.com/article/10.3390/nu15132920/s1; Table S1: Systematic Search strategy; Table S2: Data charting table; Table S3: Prisma-Scr checklist [20].

## References

[1] WHO Obesity and Overweight. https://www.who.int/news-room/fact-sheets/detail/obesity-and-overweight.

[2] Cornier M.A. (2022). A review of current guidelines for the treatment of obesity. Am. J. Manag. Care.

[3] O’Connor S.G., Boyd P., Bailey C.P., Shams-White M.M., Agurs-Collins T., Hall K., Reedy J., Sauter E.R., Czajkowski S.M. (2021). Perspective: Time-Restricted Eating Compared with Caloric Restriction: Potential Facilitators and Barriers of Long-Term Weight Loss Maintenance. Adv. Nutr..

[4] Dulloo A.G. (2021). Physiology of weight regain: Lessons from the classic Minnesota Starvation Experiment on human body composition regulation. Obes. Rev..

[5] Trexler E.T., Smith-Ryan A.E., Norton L.E. (2014). Metabolic adaptation to weight loss: Implications for the athlete. J. Int. Soc. Sports Nutr..

[6] Carreiro A.L., Dhillon J., Gordon S., Higgins K.A., Jacobs A.G., McArthur B.M., Redan B.W., Rivera R.L., Schmidt L.R., Mattes R.D. (2016). The Macronutrients, Appetite, and Energy Intake. Annu. Rev. Nutr..

[7] Garaulet M., Gómez-Abellán P., Alburquerque-Béjar J.J., Lee Y.C., Ordovás J.M., Scheer F.A.J.L. (2013). Timing of food intake predicts weight loss effectiveness. Int. J. Obes..

[8] dos Santos K.C., Olofsson C., Cunha J.P.M.C.M., Roberts F., Catrina S.B., Fex M., Ekberg N.R., Spégel P. (2022). The impact of macronutrient composition on metabolic regulation: An Islet-Centric view. Acta Physiol..

[9] Hall M.E., Cohen J.B., Ard J.D., Egan B.M., Hall J.E., Lavie C.J., Ma J., Ndumele C.E., Schauer P.R., Shimbo D. (2021). Weight-Loss Strategies for Prevention and Treatment of Hypertension: A Scientific Statement from the American Heart Association. Hypertension.

[10] Younossi Z.M., Corey K.E., Lim J.K. (2021). AGA Clinical Practice Update on Lifestyle Modification Using Diet and Exercise to Achieve Weight Loss in the Management of Nonalcoholic Fatty Liver Disease: Expert Review. Gastroenterology.

[11] Diabetes Australia (2021). Position Statement: Type 2 Diabetes Remission.

[12] Hendrix J.K., Aikens J.E., Saslow L.R. (2020). Dietary weight loss strategies for self and patients: A cross-sectional survey of female physicians. Obes. Med..

[13] Bracci E.L., Milte R., Keogh J.B. (2022). Developing and Piloting a Novel Ranking System to Assess Popular Ditery Patterns and Healthy Eating Principles. Nutrients.

[14] Ashman F., Sturgiss E., Haesler E. (2016). Exploring Self-Efficacy in Australian General Practitioners Managing Patient Obesity: A Qualitative Survey Study. Int. J. Family Med..

[15] Walsh K., Grech C., Hill K. (2019). Health advice and education given to overweight patients by primary care doctors and nurses: A scoping literature review. Prev. Med. Rep..

[16] United States Department of Agriculture (USDA) (2014). A Series of Systematic Reviews on the Relationship between Dietary Patterns and Health Outcomes.

[17] Réda A., Wassil M., Mériem M., Alexia P., Abdelmalik H., Sabine B., Nassir M. (2020). Food timing, circadian rhythm and chrononutrition: A systematic review of time-restricted eating’s effects on human health. Nutrients.

[18] Peters M., Godfrey C., McInerner P., Muinn Z., Tricco A., Khalil H., Aromataris E., Munz Z. (2020). Chapter 11: Scoping Reviews (2020 version). JBI Manual for Evidence Synthesis.

[19] Cooke A., Smith D., Booth A. (2012). Beyond PICO: The SPIDER tool for qualitative evidence synthesis. Qual. Health Res..

[20] Tricco A.C., Lillie E., Zarin W., O’Brien K.K., Colquhoun H., Levac D., Moher D., Peters M.D.J., Horsley T., Weeks L. (2018). PRISMA extension for scoping reviews (PRISMA-ScR): Checklist and explanation. Ann. Intern. Med..

[21] Nair D., Hart A. (2018). Family physicians’ perspectives on their weight loss nutrition counseling in a high obesity prevalence area. J. Am. Board Fam. Med..

[22] Wangler J., Jansky M. (2021). Attitudes, behaviours and strategies towards obesity patients in primary care: A qualitative interview study with general practitioners in Germany. Eur. J. Gen. Pract..

[23] Bąk-Sosnowska M., Skrzypulec-Plinta V. (2017). Health behaviors, health definitions, sense of coherence, and general practitioners’ attitudes towards obesity and diagnosing obesity in patients. Arch. Med. Sci..

[24] Bornhoeft K. (2018). Perceptions, Attitudes, and Behaviors of Primary Care Providers Toward Obesity Management: A Qualitative Study. J. Community Health Nurs..

[25] Dicker D., Kornboim B., Bachrach R., Shehadeh N., Potesman-Yona S., Segal-Lieberman G. (2020). ACTION-IO as a platform to understand differences in perceptions, attitudes, and behaviors of people with obesity and physicians across countries-the Israeli experience. Isr. J. Health Policy Res..

[26] Gudzune K.A., Wickham E.P., Schmidt S.L., Stanford F.C. (2021). Physicians certified by the American Board of Obesity Medicine provide evidence-based care. Clin. Obes..

[27] Mackey C., Plegue M.A., Deames M., Kittle M., Sonneville K.R., Chang T. (2018). Family physicians’ knowledge, attitudes, and behaviors regarding the weight effects of added sugar. SAGE Open Med..

[28] Mazza D., McCarthy E., Carey M., Turner L., Harris M. (2019). “90% of the time, it’s not just weight”: General practitioner and practice staff perspectives regarding the barriers and enablers to obesity guideline implementation. Obes. Res. Clin. Pract..

[29] McLeod M.R., Chionis L., Gregg B., Gianchandani R., Wolfson J.A. (2022). Knowledge and attitudes of lower Michigan primary care physicians towards dietary interventions: A cross-sectional survey. Prev. Med. Reports.

[30] Nakhoda K., Hosseinpour-Niazi S., Mirmiran P. (2021). Nutritional knowledge, attitude, and practice of general physicians toward the management of metabolic syndrome in Tehran. Shiraz E Med. J..

[31] Sharma A.M., Bélanger A., Carson V., Krah J., Langlois M., Lawlor D., Lepage S., Liu A., Macklin D.A., MacKay N. (2019). Perceptions of barriers to effective obesity management in Canada: Results from the ACTION study. Clin. Obes..

[32] VanFrank B.K., Park S., Foltz J.L., McGuire L.C., Harris D.M. (2018). Physician Characteristics Associated With Sugar-Sweetened Beverage Counseling Practices. Am. J. Health Promot..

[33] Vrkatić A., Grujičić M., Jovičić-Bata J., Novaković B. (2022). Nutritional Knowledge, Confidence, Attitudes towards Nutritional Care and Nutrition Counselling Practice among General Practitioners. Healthcare.

[34] Warr W., Aveyard P., Albury C., Nicholson B., Tudor K., Hobbs R., Roberts N., Ziebland S. (2021). A systematic review and thematic synthesis of qualitative studies exploring GPs’ and nurses’ perspectives on discussing weight with patients with overweight and obesity in primary care. Obes. Rev..

[35] Abbott S., Parretti H.M., Greenfield S. (2021). Experiences and perceptions of dietitians for obesity management: A general practice qualitative study. J. Hum. Nutr. Diet..

[36] Elliott J., Lawrence R., Minx J.C., Oladapo O.T., Ravaud P., Tendal Jeppesen B., Thomas J., Turner T., Vandvik P.O., Grimshaw J.M. (2021). Decision makers need constantly updated evidence synthesis. Nature.

