# Supplementary material for: General Practitioners’ Knowledge, Attitudes, and Practices of Dietary Advice for Weight Control in Their Overweight Patients: A Scoping Review
Source: Nutrients. 2023 Jun 27;15(13):2920. doi: 10.3390/nu15132920 (PMC10346254; doi:10.3390/nu15132920)
Supplement: Supplementary file 1 [file nutrients-15-02920-s001.zip › nutrients-2448589-supplementary.pdf]

## SUPPLEMENTARY FILE

## Supplementary Table S1. Searching strategy

**Search strategy and result from PubMed**
**PubMed [Tiab] and MeSh terms used**

Search conducted: <31.07.2022>

| | | |
|---|---|---|
| | Concept 1 terms | |
| 1 | Diet therapy[mh] OR Diet Therapy[tiab] OR Obesity[mh] OR Obesity[tiab] OR diet*[tiab] OR Nutritional counsel*[tiab] OR Nutritional advice[tiab] OR Nutritional intervention[tiab] OR Nutritional education[tiab] OR Nutritional approach[tiab] OR Lifestyle counsel*[tiab] OR Lifestyle advice[tiab] OR Lifestyle education[tiab] OR weight control[tiab] OR weight loss[tiab] OR weight management[tiab] OR weight intervention[tiab] OR weight maintenance[tiab] OR Overweight[tiab] OR health advice[tiab] | 178,991 |
| | Concept 2 terms | |
| 2 | General Practitioners[mh] OR General Practitioner[tiab] OR Physicians, Family[mh] OR Physicians, family[tiab] OR Physicians, Primary Care[mh] OR Physicians, Primary Care [tiab] OR GP[tiab] OR Clinician[tiab] OR Physician[tiab] OR Family doctor[tiab] OR Family practitioner[tiab] OR primary care practitioner[tiab] | 70,439 |
| | Concept 3 terms | |
| 3 | Physician’s Role[mh] OR Physician’s Role[tiab] OR Health Knowledge, Attitudes, Practice[mh] OR Health Knowledge, Attitudes, Practice[tiab] | 33,962 |
| | Combined terms | |
| | **1 AND 2 AND 3** | **143** |
| | **Filters**<br>**Start Date: 1 January 2017**<br>**Language: English**<br>**Species: Human** | |

**Search strategy and result from CINAHL**
**CINAHL and MeSH terms used**

Search conducted: <31.07.2022>

| | | |
|---|---|---|
| | Concept 1 terms | |
| 1 | (MH "Diet Therapy") OR (MH "Obesity") OR diet* OR “Nutritional counsel*” OR “Nutritional intervention” OR “Nutrition education” OR “Nutritional approach” OR “Lifestyle counsel*” OR “Lifestyle advice” OR “Lifestyle education” OR “weight loss” OR “weight management” OR “weight intervention” OR “weight maintenance” OR “Obesity” OR “Obese” OR “Overweight” | 60,085 |
| | Concept 2 terms | |
| 2 | (MH"Physician,Family") OR “General Practitioners” OR “GP” OR “Clinician” OR “Physician” OR “Family doctor” OR “Family practitioner” OR “primary care practitioner” | 28,084 |
| | Concept 3 terms | |

| | | |
|---|---|---|
| 3 | (MH "Attitude of Health Personnel") OR (MH "Physician's Role") OR "Health Knowledge, Attitudes, Practice" OR "KAP" | 11,852 |
| | Combined terms | |
| | **1 AND 2 AND 3** | **79** |
| | **Filters**<br>**Start Date: 1 January 2017**<br>**Language: English**<br>**Species: Human** | |

**Search strategy and result from Ovid EMBASE**
**EMBASE (Ovid)**

**Embase Classic + Embase**

Search conducted: <31.07.2022>

| | | |
|---|---|---|
| | Concept 1 terms | |
| 1 | diet therapy.mp. OR exp diet therapy/ OR obesity.mp. OR exp obesity/ OR exp nutritional counseling/ or nutritional counseling.mp. OR exp diet/ or diet.mp. OR nutritional advice.mp. OR nutritional intervention.mp. OR nutritional education.mp. OR Lifestyle counsel*.mp. OR Lifestyle advice.mp. OR lifestyle education.mp. OR weight control.mp. OR weight intervention.mp. OR Weight maintenance.mp. Or Nutrition.mp | 401,318 |
| | Concept 2 terms | |
| 2 | General practitioner.mp. OR exp general practitioner/ OR primary care practitioner.mp. OR Family Physician.mp. OR Physicians, family.mp. OR Physician.mp. | 172,594 |
| | Concept 3 terms | |
| 3 | Physicians role.mp. OR exp physician attitude/ OR Health Knowledge, Attitudes, Practice.mp. OR exp attitude to health/ OR Health Knowledge, Attitudes, Practice.mp. | 38,364 |
| | Combined terms | |
| | **1 AND 2 AND 3** | **484** |
| | **Filters**<br>**Start Date: 1 January 2017- Current**<br>**Language: English**<br>**Species: Human** | |

**Search strategy and result from Ovid MEDLINE**
**Ovid MEDLINE**

Search conducted: <31.07.2022>

| | | |
|---|---|---|
| | Concept 1 terms | |
| 1 | diet therapy.mp. OR exp diet therapy/ OR obesity.mp. OR exp obesity/ OR nutritional counseling.mp. OR exp diet/ or diet.mp. OR nutritional advice.mp. OR nutritional intervention.mp. OR nutritional education.mp. OR Lifestyle counsel*.mp. OR Lifestyle advice.mp. OR lifestyle | 190,345 |

| | | |
|---|---|---|
| | education.mp. OR weight control.mp. OR weight intervention.mp. OR Weight maintenance.mp. Or Nutrition.mp OR Overweight.mp. OR Overweight/ or health advice.mp. | |
| | Concept 2 terms | |
| 2 | General practitioner.mp. OR exp general practitioners/ OR Physicians/ OR physicians.mp Or Family physicians.mp. or Physicians, family/ OR Physicians primary care.mp. or Physicians, Primary Care/ OR primary care practitioner.mp. OR Physician, primary care.mp. OR Physicians, Primary Care/ Or Doctor.mp. | 92,234 |
| | Concept 3 terms | |
| 3 | Physicians role.mp. or Physician's Role/ or Health Knowledge, Attitudes, Practice.mp. or Health Knowledge, Attitudes, Practice/ or Attitude to Health.mp. or Attitude to Health/ or Health attitudes.mp. | 58,228 |
| | Combined terms | |
| | **1 AND 2 AND 3** | **396** |
| | **Filters**<br>**Start Date: 1 January 2017-current**<br>**Language: English**<br>**Species: Human** | |

**Supplementary Table S2. Data Charting Table**

| No | Author, Year | Aim/ Objective | Location/ Setting/ Design | Participants | Instrument | Dietary Advice Reported | Aspect of Diet | Findings on Knowledge | Findings on Attitude | Findings on Practice | Conclusion / Reccomendation |
|---|---|---|---|---|---|---|---|---|---|---|---|
| | surname of first author, year of publication | | country \| place (hospital, primary care, doctor's office, etc) - rural/urban/remote? \| survey/rct/qualitative/mixed method | no of participants, inclusion/exclusion criteria | FGD/interview/questionnaire/etc - incl selfchecklist / guideline based/open ended etc. | What kind nutrition advice discussed (calorie counting, tracking, elimination diet, fasting, fruit&vegs, etc) | Quality / quantity / timing / guidelines | Yes/No, How? | Yes/No, How? | Yes/No, How? | |

## Supplementary Table S3. PRISMA-Scr Checklist

**Preferred Reporting Items for Systematic reviews and Meta-Analyses extension for Scoping Reviews (PRISMA-ScR) Checklist [20].**

| SECTION | ITEM | PRISMA-ScR CHECKLIST ITEM | REPORTED ON PAGE # |
|---|---|---|---|
| **TITLE** | | | |
| Title | 1 | Identify the report as a scoping review. | 1 |
| **ABSTRACT** | | | |
| Structured summary | 2 | Provide a structured summary that includes (as applicable): background, objectives, eligibility criteria, sources of evidence, charting methods, results, and conclusions that relate to the review questions and objectives. | 1 |
| **INTRODUCTION** | | | |
| Rationale | 3 | Describe the rationale for the review in the context of what is already known. Explain why the review questions/objectives lend themselves to a scoping review approach. | 1-2 |
| Objectives | 4 | Provide an explicit statement of the questions and objectives being addressed with reference to their key elements (e.g., population or participants, concepts, and context) or other relevant key elements used to conceptualize the review questions and/or objectives. | 2 |
| **METHODS** | | | |
| Protocol and registration | 5 | Indicate whether a review protocol exists; state if and where it can be accessed (e.g., a Web address); and if available, provide registration information, including the registration number. | 3 |
| Eligibility criteria | 6 | Specify characteristics of the sources of evidence used as eligibility criteria (e.g., years considered, language, and publication status), and provide a rationale. | 3-4 |
| Information sources* | 7 | Describe all information sources in the search (e.g., databases with dates of coverage and contact with authors to identify additional sources), as well as the date the most recent search was executed. | 3 |
| Search | 8 | Present the full electronic search strategy for at least 1 database, including any limits used, such that it could be repeated. | 3 and Appendix 1 |
| Selection of sources of evidence† | 9 | State the process for selecting sources of evidence (i.e., screening and eligibility) included in the scoping review. | 4 |
| Data charting process‡ | 10 | Describe the methods of charting data from the included sources of evidence (e.g., calibrated forms or forms that have been tested by the team before their use, and whether data charting was done independently or in duplicate) and any processes for obtaining and confirming data from investigators. | 4 |
| Data items | 11 | List and define all variables for which data were sought and any assumptions and simplifications made. | Appendix 2 |
| Critical appraisal of individual sources of evidence§ | 12 | If done, provide a rationale for conducting a critical appraisal of included sources of evidence; describe the methods used and how this information was used in any data synthesis (if appropriate). | N/A |

| SECTION | ITEM | PRISMA-ScR CHECKLIST ITEM | REPORTED ON PAGE # |
|---|---|---|---|
| Synthesis of results | 13 | Describe the methods of handling and summarizing the data that were charted. | 4 |
| **RESULTS** | | | |
| Selection of sources of evidence | 14 | Give numbers of sources of evidence screened, assessed for eligibility, and included in the review, with reasons for exclusions at each stage, ideally using a flow diagram. | 4-5 |
| Characteristics of sources of evidence | 15 | For each source of evidence, present characteristics for which data were charted and provide the citations. | 6 |
| Critical appraisal within sources of evidence | 16 | If done, present data on critical appraisal of included sources of evidence (see item 12). | N/A |
| Results of individual sources of evidence | 17 | For each included source of evidence, present the relevant data that were charted that relate to the review questions and objectives. | 8-12 |
| Synthesis of results | 18 | Summarize and/or present the charting results as they relate to the review questions and objectives. | 13-14 |
| **DISCUSSION** | | | |
| Summary of evidence | 19 | Summarize the main results (including an overview of concepts, themes, and types of evidence available), link to the review questions and objectives, and consider the relevance to key groups. | 15 |
| Limitations | 20 | Discuss the limitations of the scoping review process. | 16 |
| Conclusions | 21 | Provide a general interpretation of the results with respect to the review questions and objectives, as well as potential implications and/or next steps. | 17 |
| **FUNDING** | | | |
| Funding | 22 | Describe sources of funding for the included sources of evidence, as well as sources of funding for the scoping review. Describe the role of the funders of the scoping review. | 17 |

JBI = Joanna Briggs Institute; PRISMA-ScR = Preferred Reporting Items for Systematic reviews and Meta-Analyses extension for Scoping Reviews.

*From:* 20. Tricco, A.C.; Lillie, E.; Zarin, W.; O'Brien, K.K.; Colquhoun, H.; Levac, D.; Moher, D.; Peters, M.D.J.; Horsley, T.; Weeks, L.; et al. PRISMA extension for scoping reviews (PRISMA-ScR): Checklist and explanation. Ann. Intern. Med. 2018, 169, 467–473. https://doi.org/10.7326/M18-0850
